# Effective inhibition of T95 steel corrosion in 15 wt% HCl solution by aspartame, potassium iodide, and sodium dodecyl sulphate mixture

**DOI:** 10.1038/s41598-023-40354-8

**Published:** 2023-08-11

**Authors:** Moses M. Solomon

**Affiliations:** Department of Chemical and Environmental Engineering, University of Nottingham Ningbo China, Ningbo, China

**Keywords:** Chemistry, Materials science

## Abstract

Sustainable development goal 12 advocates the production and consumption of green and sustainable commodities. As such, pressure is mounting on the oil and gas industries for a paradigm shift. This work explores the potential of aspartame (a derivative of aspartic acid and phenylalanine) based formulation as a green inhibitor. The inhibiting effect of aspartame alone and in combination with potassium iodide (KI) or sodium dodecyl sulphate (SDS) or both on T95 steel in 15 wt% HCl solution at 60–90 °C is investigated using weight loss, electrochemical, and surface analysis techniques. The results show severe metal corrosion especially at 90 °C with a corrosion rate (*v*) of 186.37 mm/y. Aspartame inhibits corrosion and its inhibition efficiency (η) increases with an increase in temperature. At 6.80 mM, η of 86% is obtained at 90 °C. The addition of SDS to aspartame produces an antagonistic effect. A KI-aspartame mixture produces an antagonistic effect at 60 °C and 70 °C but a synergistic effect at 80 °C and 90 °C. There is a strong synergy when aspartame (6.80 mM), KI (1 mM), and SDS (1 mM) are mixed especially at higher temperatures. The mixture reduces *v* from 186.37 to 14.35 mm/y, protecting the metal surface by 92% at 90 °C. The mixture can be considered an acidizing corrosion inhibitor.

## Introduction

As the campaigns for green and sustainable chemical manufacturing and usage intensify, the oil and gas industries are facing mounting transition pressure because of the peculiarity of the sector. Every operational stage in the sector presents a difficult terrain to reach the new ‘green world’. For example, the current chemical corrosion inhibitor compositions are based on primary amines, quaternary salts of amines, imidazolines, surfactants, intensifiers, etc.^1,2^. The package is designed to exhibit surfactant-like properties of film-forming and persistence^3^ under high-flow conditions and excellent inhibition performance under various field conditions. The organic-based formulation was a milestone achievement and was highly celebrated as they were seen as the perfect replacement for the poor-performing sodium arsenite and sodium ferrocyanide^2^ although limited at high-temperature application^4,5^ which many research works^6^ have attempted to address. However, the concern over their hazardous contribution to the environment^7^ relating to marine toxicity (inherent toxic property of most nitrogen-based compounds^8^) and non-biodegradation is making them less acceptable for use in highly regulated offshore environments^9^. Corrosion inhibitor scientists are now faced with the dilemma of producing corrosion inhibitors that are highly effective and persist under flow conditions but biodegradable and environmentally acceptable.

**Figure 1:**
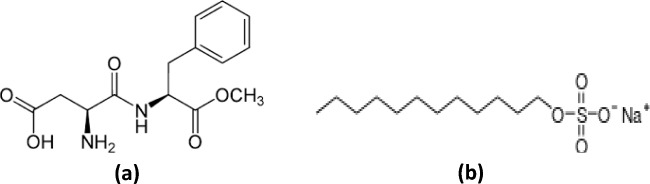
Chemical structure of (**a**) aspartame and (**b**) sodium dodecyl sulphate.

Aspartame (Fig. 1a), (*N*-(l-α-Aspartyl)-l-phenylalanine) is a derivative of aspartic acid and phenylalanine with the United States Food and Drug Administration (FDA) approval for use as an artificial sweetener by the food and drug industries^10^. It is cost-effective, has LD50 (oral) of 10,000 mg/kg^11^, and contains the heteroatoms O and N in its molecule as possible adsorption centres (Fig. 1a). Besides the aforementioned properties, the interest in aspartame in this study also stems from its high -melting point of 246–247 °C. The melting point of a molecule is an important parameter to consider when designing for high-temperature applications such as oil well acidizing. In a previous research work^12^, it was found that aspartame is a highly promising acidizing corrosion inhibitor. Its inhibition efficiency increased with an increase in temperature reaching 86% at 90 °C. This work is an extension of the previous and is aimed at identifying compounds that could act as intensifiers to boost the corrosion inhibition property of aspartame for T95 steel in a strong acid medium (15 wt% HCl) at high temperatures (60–90 °C). Electrochemical frequency modulation (EFM) and weight loss (WL) techniques are used to revalidate the previous results^12^. The effect of the addition of potassium iodide or sodium dodecyl sulphate (SDS, Fig. 1b) or both on the inhibition performance of aspartame is studied using the WL, electrochemical impedance spectroscopy (EIS), potentiodynamic polarization (PDP), scanning electron microscope (SEM), and optical profilometer (OP).

The T95-2 steel grade (for convenience, T95 is adopted in the manuscript) is known for its high strength and resistance to sulphide stress corrosion making them the preferred material for oil and gas well tubing^13,14^. Nevertheless, well-stimulation activities such as acidizing whereby a strong acid solution is pumped into a reservoir to dissolve flow channel barriers^15^ affect the corrosion resistance property of the tubing negatively. The normal practice has been the fortification of the acidizing solution with an effective corrosion inhibitor^15^ which is the basis for corrosion inhibitor research in acid concentrations of 15–28 wt%. Subramania et al.^16^ investigated the inhibitive effect of 1-cinnamylidine-3-thiocarbohydrazide (CTCH) and 1,1′-dicinnamylidine-3-thiocarbohydrazide (DCTCH) on carbon steel dissolution in 15 wt% HCl solution. At 110 °C, 1500 ppm of CTCH was found to reduce the corrosion rate of the metal from 14,417.0 to 255.96 mm/y and protected the metal surface by 98.2%. At the same temperature and concentration, a corrosion rate and inhibition efficiency of 140.31 mm/y and 99.0%, respectively was recorded for DCTCH from the weight loss technique. A formulation consisting of 1.0 mM N-acetyl cysteine + 10^−5^ M glutathione + 10^−5^ M KI was reported to afford an inhibition efficiency of 91.4% at 90 °C on X80 steel in 15 wt% HCl solution^17^. Dicinnamylidene acetone, disalicylidene acetone, divanillidene acetone^18^, *N*-(2-(2-tridecyl-4,5-dihydro-1H-imidazol-1-yl)ethyl) tetradecanamide^19^, (E)-5-amino-3-(4-methoxyphenyl)-*N*′-(1-(4-methoxyphenyl)ethylidene)-1H-pyrazole-4-carbohydrazide, (E)-5-amino-*N*′-(4-chlorobenzylidene)-3-(4-chlorophenyl)-1H-pyrazole-4-carbohydrazide^20^, among others, have been reported as corrosion inhibitors for carbon steel in 15 wt% HCl solution. As earlier mentioned, environmental concerns and the complicated synthesis procedures for some of these compounds are the limitations. This communication thus showcases aspartame-based formulation as a green and sustainable corrosion inhibitor for the acidizing process.

## Experimental aspect

### Materials and reagents preparation

Aspartame (CAS No: 22839-47-0, molar mass: 294.3 g/mol), KI, SDS, HCl (37%), and ethanol were purchased from Merck company (USA). They were of analytical grade. T95 steel grade with composition given in Uzoma et al.^12^ was sourced from Wuhan Corrtest Instruments Corp., Ltd, China. The dimension of the samples used for weight loss studies was 2 × 2 × 1 and the surface area (*A*) was calculated following Eq. (1)^21^.

1 $$A=2\left(wl+dl+dw\right)$$

where *w* is the width (cm), *d* is the thickness (cm) and *l* is the length (cm). For samples used for the electrochemical studies, the exposed surface area was 1 cm^2^. The surface pre-treatment followed the procedure listed in ASTM G1-90^22^. Mechanical surface abrasion was accomplished on the EcoMet metallographic abrasion instrument (Buehler Co., USA) with sandpapers of grit size ranging from #120 to #2000. At room temperature, aspartame is not readily soluble in water. Hence, the stock solution (339.79 mM) was prepared by dissolving the appropriate amount in a 1:1 ratio mixture (20 mL) of ethanol and distilled water. The experimental concentrations of aspartame (1.70 mM and 6.80 mM) were obtained by serial dilution of the stock solution with 15 wt% of HCl solution. The concentrations of KI and SDS considered in the study are 1, 3, and 5 mM.

### Weight loss experiments

Before the experiment, the weight of the prepared samples was measured and recorded as the initial weight. Sealed reaction bottles (150 cm^3^ capacity) with appropriate label (i.e., blank, 1.70 mM aspartame, etc.) was filled with 100 cm^3^ of the respective solutions and placed on a digital thermostatic water bath for the solutions to attain the studied temperature. The temperatures studied were 60 °C, 70 °C, 80 °C, and 90 °C. A thermometer was used to confirm that the temperature of the bottles’ solutions was as expected. Thereafter, the prepared samples, in triplicate, were freely suspended in each of the bottles with the help of a thread, the bottles were corked, and allowed to stand at the studied temperature for 4 h. Subsequently, the corroded samples were retrieved, immersed in a pickling solution (50 g NaOH + 200 g granulated Zn dust made up to 1000 mL with distilled water^22^ for 10 min, gently scrubbed under running water and acetone, dried with warm air, and re-weighed. The weight loss (g), corrosion rate (mm/y), and inhibition efficiency (η_WL_%) were calculated as^23^:

2 $$WL={W}_{i}-{W}_{f}$$

3 $$v=\frac{87600\times WL}{\rho At}$$

4 $${\eta }_{\mathrm{WL}}\%=\frac{{v}_{0}-{v}_{i}}{{v}_{0}}\times 100$$

where $WL$ is the average weight loss (g), ${W}_{i}$ is the weight of the sample before corrosion studies (g), ${W}_{f}$ is the weight of the sample after the corrosion studies, $\rho$ is density in g/cm^3^, *t* is the immersion time (h), ${v}_{0}\mathrm{ and }{v}_{i}$ are the corrosion rates of the specimen in the blank and inhibited solutions, respectively.

### Electrochemical studies

The electrochemical wet corrosion tests, namely EFM, EIS, and PDP were conducted in a workstation consisting of a T95 steel working electrode, an Ag|AgCl (3 M KCl) reference electrode, and a graphite rod counter electrode via a Gamry Potentiostat/Galvanostat/ZRA Reference 600 instrument. After attaining open circuit potential stability (3600 s of delay), the EIS data were collected at OCP with ± 10 mV perturbation potential amplitude in the frequency region of 0.01 Hz to 100,000 Hz. The analysis of these data was accomplished with Echem software. The η_EIS_ was estimated using Eq. (5)^24^.

5 $${\eta }_{\mathrm{EIS}}\%=\frac{{R}_{p(1)}-{R}_{p(0)}}{{R}_{p(1)}}\times 100$$

6 $${R}_{\mathrm{p}}={R}_{\mathrm{f}}+{R}_{\mathrm{ct}}$$

where ${R}_{p(0)}$ and ${R}_{p(1)}$ are the polarization resistances in corrodent without and with additives, respectively, ${R}_{\mathrm{f}}$ is the surface oxide or adsorbed film resistance, and ${R}_{\mathrm{ct}}$ is the charge transfer resistance^24^.

EFM data were collected by setting the base frequency, the applied amplitude, and the multiplier A and B frequencies at 1 Hz, 10 mV, 2 Hz, and 5 Hz, respectively. A sinusoidal waveform of 32 cycles was recorded per measurement. Finally, the working electrode was polarized at the potential of ± 250 mV from OCP using a scan rate of 0.2 mVs^−1^ to obtain the PDP data. The corrosion current density (*i*_corr_) was used for the estimation of both ${\eta }_{\mathrm{EFM}}$ and η_PDP_ following Eq. (7).

7 $${\eta }_{\mathrm{EFM or PDP}}\%=\frac{{i}_{corr}^{blank}-{i}_{corr}^{inhibited}}{{i}_{corr}^{blank}}\times 100$$

### Surface examination

The morphology and the elemental composition of the T95 samples before and after corrosion in 15 wt% HCl solution without and with additives for 4 h at 60 °C and 90 °C were scrutinized with a JEOL JSM-6610 LV scanning electron microscope (SEM) equipped with an energy-dispersive X-ray spectroscopy (EDX) detector. A 5420 atomic force microscope (N9498S, Agilent Technologies, U.K.) operated in the contact mode under normal conditions was used for surface roughness characterisation. A 3D optical profilometer (Contour GT-K, Bruker Nano GmBH, Berlin, Germany) was utilised to probe the 3D topographies of the T95 steel samples before and after 4 h of exposure to unprotected and protected 15 wt% HCl solutions at 60 °C and 90 °C.

### Consent for publication

The author has the consent of the University of Nottingham Ningbo China to publish this work.

## Results and discussion

### Validation of the corrosion inhibition by aspartame

**Figure 2:**
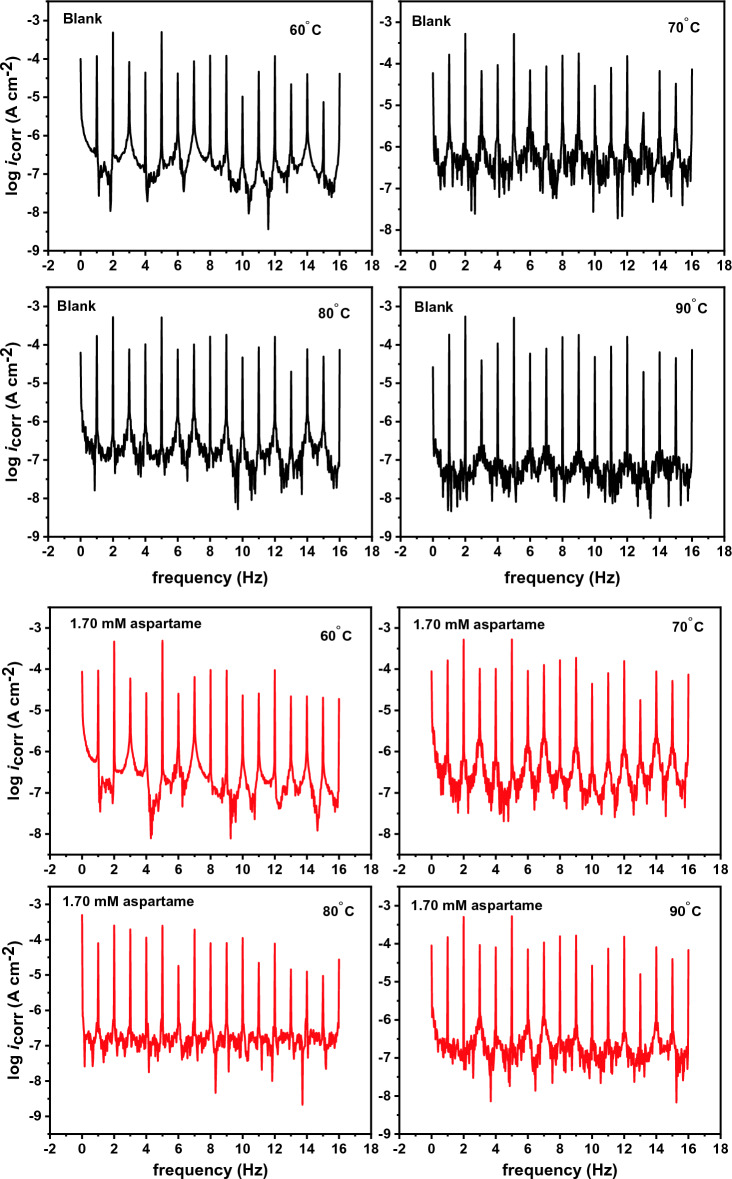

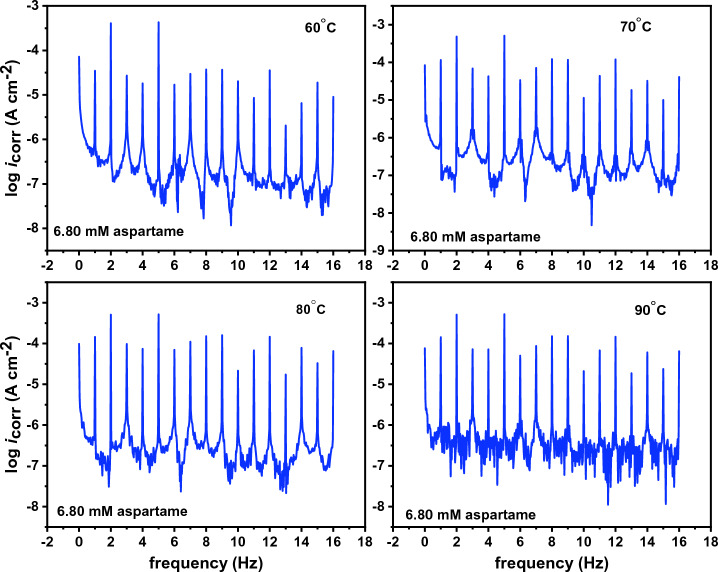
Intermodulation spectrum recorded for T95 corrosion in 15 wt% HCl solution in the absence and presence of selected concentrations of aspartame at different temperatures.

The EIS and PDP techniques were previously used to study the inhibition performance of aspartame for T95 steel in 15 wt% HCl solution at 60–90 °C^12^. Before proceeding to the synergistic inhibition studies which is the main focus of this work, the previous results were validated using the classical WL and EFM techniques. The EFM technique, in particular, was opted for because of the internal data validation mechanism, the so-called causality factor^23^. As is known^25^, the ideal value of causality 2 (CF2) and 3 (CF3) factors is 2 and 3, respectively. Hence, experimentally obtained values must fall within the 0–2 and 0–3 range for the results to be meaningful. Figure 2 shows EFM spectra for T95 in 15 wt% HCl solution in the absence and presence of 1.70 mM or 6.80 mM of aspartame at 60 °C, 70 °C, 80 °C, and 90 °C. The *i*_corr_, anodic and cathodic slopes (*β*_a_ and *β*_c_), CF2, and CF3 values derived from the analysis of the EFM data are given in Table 1. η_EFM_ as earlier stated was computed using Eq. (7).

**Table 1:** Electrochemical frequency modulation (EFM) and Weight loss (WL) data for T95 corrosion in 15 wt% HCl solution in the absence and presence of selected concentrations of aspartame at different temperatures.

Conc. (mM)	EFM						WL	
	*i*_corr_ (µA cm^−2^)	*β*_a_ (mV dec^−1^)	*β*_c_ (mV dec^−1^)	CF2	CF3	η_EFM_	*v* (mm/y)	η_WL_%
60 °C								
0	182.9	21.3	27.4	2.114	3.045	-	26.20 ± 0.17	-
1.70	150.8	23.9	29.5	2.105	3.109	16	21.68 ± 0.17	17
6.80	98.7	36.6	43.2	1.486	2.015	46	13.18 ± 1.73	50
70 °C								
0	167.7	19.1	23.1	1.254	3.171	-	60.20 ± 3.47	-
1.70	118.2	18.3	24.3	1.564	2.352	30	42.32 ± 0.17	30
6.80	80.3	21.7	26.7	2.094	3.416	52	25.50 ± 0.17	58
80 °C								
0	166.5	18.6	23.1	1.19	2.722	-	106.17 ± 1.73	-
1.70	101.4	20.0	41.8	1.715	3.114	39	62.63 ± 0.17	41
6.80	63.1	19.3	25.3	2.167	2.916	62	39.38 ± 0.17	63
90 °C								
0	165	19.0	21.9	2.184	3.287	-	149.54 ± 8.69	-
1.70	61	19.1	24.6	1.866	2.804	63	42.33 ± 1.73	72
6.80	42.5	19.8	24.4	1.720	3.186	74	21.34 ± 1.73	86

In Table 1, the CF2 and CF3 are in the acceptable region, indicating a favourable causality between perturbation and signal^23^ and reliable results. The spectra in Fig. 2 all contain strong harmonic and intermodulation signals as well as weak background noise signals. A close inspection of the EFM spectra reveals that the harmonic and intermodulation signals are suppressed with increasing frequency, especially in the aspartame-containing systems. This, according to Liu et al.^23^ is due to a decrease in *i*_corr_. An examination of Table 1 discloses that the *i*_corr_ value of the T95 steel specimen in the blank acid solution slightly decreased from 182.9 at 60 °C to 167.7, 166.5, and 165.0 µA cm^−2^ at 70 °C, 80 °C, and 90 °C, respectively indicating the slight inhibitive effect of the corrosion products deposited on the surface. The *i*_corr_ value for the aspartame-inhibited systems noticeably reduced and the extent of reduction is dependent on concentration and temperature. It decreases with increasing concentration and temperature. The least *i*_corr_ value is observed at 90 °C where it decreased from 165.0 to 42.5 µA cm^−2^ in the 6.80 mM aspartame-containing solution. Consequently, the inhibition efficiency of aspartame increases with an increase in concentration and temperature. A similar trend was observed in the previous studies^12^. In the report, inhibition efficiency of 47%, 51%, 62%, and 85% were reported for 6.80 mM of aspartame at 60 °C, 70 °C, 80 °C, and 90 °C, respectively from the EIS technique. In the current work, the η_EFM_ for 6.80 mM aspartame is 46%, 52%, 62%, and 74%, respectively at 60 °C, 70 °C, 80 °C, and 90 °C. The results from the two techniques are in excellent agreement and therefore validate the inhibitive performance of aspartame in the studied medium. More so, a mixed-type inhibition mechanism was proposed for aspartame. A close look at the *β*_a_ and *β*_c_ values in Table 1 reveals that there is no mark difference. For instance, at 80 °C, the *β*_a_ value is 21.7 mV dec^−1^ while the *β*_c_ value is 26.7 mV dec^−1^ for the 6.80 mM aspartame-inhibited system. This again ratifies the mixed-type corrosion mechanism. Furthermore, the *v* and η_WL_ values (Table 1) from the weight loss method follow the trend spotted for *i*_corr_ and η_EFM_. However, because η_WL_ is from the measurement of average corrosion rate^26^, while η_EFM_ is from the instantaneous corrosion rate measurement, η_WL_ is slightly larger than η_EFM_.The increase of η_EFM_ and η_WL_ with a rise in the temperature of the solution indicates the chemisorption of aspartame molecules on the steel surface^27^. This could be possible through the donation of electron pairs from the heteroatoms (N and O) of aspartame (Fig. 1a) to the low-lying empty d-orbital ([Ar]3d^6^) of Fe^2+^^27^.

### Effect of the addition of KI and SDS on aspartame inhibition

#### Studies on individual inhibition performance by additives

The weight loss technique was adopted to study the individual inhibition performance by KI and SDS against T95 corrosion in 15 wt% HCl solution at a temperature range of 60–90 °C. To be guided in the choice of the concentration selection for synergistic studies, three concentrations, namely 1 mM, 3 mM, and 5 mM were studied. The calculated value of WL, *v*, surface coverage (θ), and η_WL_ for T95 corrosion in the corrodent free of and containing the chosen concentrations of KI or SDS at various temperatures are summarized in Table 2. As should be expected, WL and *v* in both uninhibited and inhibited systems increase with a rise in temperature because of the corresponding increase in the kinetic energy of the attacking species^28^. Both KI and SDS exhibit corrosion-inhibiting properties. The WL and *v* values can be seen to reduce in the additives-containing systems at all temperatures. The θ and η_WL_ values indicate that the additives adsorb on the steel surface, cover some portions, and offer a certain degree of protection against the metal surface corrosion. The η_WL_ value of the additives which is in the range of 35–10% is seen to decrease with an increase in concentration and temperature. The best inhibition performance is obtained for the 1 mM concentration and at 60 °C.

**Table 2:** Weight loss (WL), corrosion rate (v), surface coverage (θ), and inhibition efficiency (η_WL_) for T95 corrosion in 15 wt% HCl solution in the absence and presence of various concentrations of potassium iodide (KI) and sodium dodecyl sulphate (SDS) at different temperatures from weight loss technique.

Conc	WL ± S.D (g)	(*v* ± S.D) (mm/y)	θ	η_WL_%	WL ± S.D (g)	(*v* ± S.D) (mm/y)	θ	η_WL_%
	60 °C				70 °C			
Blank	0.15 ± 0.04	26.20 ± 0.17	–	–	0.35 ± 0.02	60.20 ± 3.47	–	–
1 mM KI	0.10 ± 0.01	17.00 ± 0.17	0.35	35	0.24 ± 0.02	41.63 ± 0.17	0.32	32
3 mM KI	0.11 ± 0.01	18.22 ± 2.26	0.31	31	0.25 ± 0.20	43.54 ± 2.26	0.28	28
5 mM KI	0.11 ± 0.02	19.43 ± 3.99	0.26	26	0.27 ± 0.01	46.14 ± 0.87	0.23	23
1 mM SDS	0.11 ± 0.01	18.22 ± 1.21	0.30	30	0.26 ± 0.01	44.24 ± 1.73	0.26	26
3 mM SDS	0.12 ± 0.01	20.30 ± 0.52	0.22	22	0.28 ± 0.08	48.75 ± 4.39	0.19	19
5 mM SDS	0.13 ± 0.02	22.21 ± 0.35	0.15	15	0.30 ± 0.02	51.18 ± 3.64	0.15	15
	80 °C				90 °C			
Blank	0.61 ± 0.01	106.17 ± 1.73	–	–	0.86 ± 0.05	149.54 ± 8.67	–	–
1 mM KI	0.43 ± 0.06	74.60 ± 6.41	0.30	30	0.62 ± 0.06	107.56 ± 9.41	0.28	28
3 mM KI	0.45 ± 0.06	78.07 ± 5.20	0.26	26	0.67 ± 0.02	116.23 ± 3.47	0.23	23
5 mM KI	0.49 ± 0.03	85.00 ± 5.20	0.20	20	0.71 ± 0.02	123.17 ± 3.47	0.17	17
1 mM SDS	0.48 ± 0.03	83.27 ± 5.20	0.22	22	0.67 ± 0.01	116.23 ± 1.73	0.22	22
3 mM SDS	0.53 ± 0.08	91.94 ± 7.88	0.13	13	0.69 ± 0.01	119.70 ± 1.73	0.19	19
5 mM SDS	0.52 ± 0.05	90.21 ± 8.67	0.15	15	0.71 ± 0.08	123.17 ± 9.88	0.17	17

KI, potassium iodide; SDS, Sodium dodecyl sulphate.

It has been experimentally proven that iodide ions (I^−^) exhibit some degree of inhibition against Fe corrosion in acid media due to its specific adsorption^29–31^. Farag and Hegazy^29^ reported an inhibition efficiency of 82.7% for the protection of carbon steel against corrosion in 0.5 M H_2_SO_4_ solution by 0.05 M KI at 25 °C. Haruna et al.^32^ reported an inhibition efficiency value of 70.42% for 2.5 w/v% KI against the corrosion of X60 carbon steel in 15 wt% HCl at 25 °C. Feng et al.^33^ however, observed a fluctuating inhibition performance by iodide ions with increasing concentrations typical of the observation in this work (Table 2). In an acid solution with dissolved oxygen and a potential of about 0.625 V_SHE_ or 0.381 V_SCE_, I^−^ ions are oxidized to ${\mathrm{I}}_{3}^{-}$ ions (Eqs. 8–10)^33^.

8 $${2\mathrm{I}}^{-}\to {\mathrm{I}}_{2}+2{e}^{-}$$

9 $${{\mathrm{I}}_{2}+\mathrm{ I}}^{-}\leftrightarrow {\mathrm{I}}_{3}^{-}$$

10 $${3\mathrm{I}}^{-}\to {\mathrm{I}}_{3}^{-}+2{e}^{-}$$

Feng et al.^33^ noted that the formation of triiodide ion (${\mathrm{I}}_{3}^{-}$) rather lowered the inhibition efficiency of I^−^. If the ${\mathrm{I}}_{3}^{-}$ species do not provide as much inhibition as the I^-^ ions, it means fewer I^-^ ions would be available for adsorption and protection of the steel surface at higher concentrations of KI and temperatures. The oxidation of I^-^ ions to ${\mathrm{I}}_{3}^{-}$ ions should increase as the temperature of the system is increased. More I^-^ ions should be available for oxidation to ${\mathrm{I}}_{3}^{-}$ ions for higher concentrations. This may be the reason for the decreasing trend in the inhibition efficiency of KI with increasing concentration and temperature. It should also be mentioned that the inhibition of KI significantly depreciates in 15 wt% HCl at 60–90 °C compared to the value reported for the same corrosive medium but at lower temperatures^32^. This further alludes to the possibility of ${\mathrm{I}}_{3}^{-}$ ions being less inhibiting than I^-^ ions. Investigating the inhibition property of various species of iodide ions will be interesting.

For the SDS, Tan et al.^34^, based on molecular simulation results proposed the mechanism of inhibition of carbon steel dissolution in 1 M HCl at 25 °C by SDS to originate from self-assembled hydrophobic barrier formation on the metal surface due to a connection between the polar sulfonic acid group to the Fe atoms and the spreading of the long alkyl chain in solution. The authors, notwithstanding noticed that there was no significant change in the inhibition efficiency value upon an increase in SDS concentration from 1 mM (68.6%) to 5.0 mM (69.5%). It is rational to attribute the observed decline in inhibition efficiency of SDS with increasing concentration and temperature to system saturation and detachment, respectively.

#### Studies on collective inhibition performance by additives with aspartame

##### WL studies

The 1 mM concentration of KI and SDS was chosen for synergistic inhibition studies with aspartame since it exhibited the highest inhibiting effect. The results of the synergistic studies from the weight loss technique are given in Table 3. Formulation denotes a mixture of 6.80 mM aspartame with 1 mM of KI and SDS. An interesting observation is made in Table 3. The addition of KI to aspartame enhances inhibitive performance at all temperatures while the reverse is observed for the aspartame + SDS combination. For instance, the η_WL_ of aspartame at 60 °C, 70 °C, 80 °C, and 90 °C is 50%, 58%, 63%, and 86%, respectively (Table 1). With a combination with 1 mM KI, the performance improves to 59%, 66%, 69%, and 90% at 60 °C, 70 °C, 80 °C, and 90 °C, respectively. However, a combination with 1 mM SDS sees the inhibiting performance depreciates to 41% and 58% at 60 °C and 80 °C, respectively, and remains the same at 70 and 90 °C (Table 3). This can be explained by the fact that I^-^ ions preferentially adsorbed on the anodic area, create a negative substrate surface that electrostatically attracts protonated aspartame molecules. However, on the surface, molecular interactions occasioned by the chemisorption process involving electron donation from the aspartame heteroatoms and the acceptance by the 3d-orbitals of Fe (in steel) ensured. Thus, I^-^ ions are involved in the stabilisation of the adsorbed aspartame molecules^29,35^. In the case of SDS, according to Tan et al.^34^, the adsorption of SDS molecules on a steel surface in an HCl solution occurs through the S and O atoms of the polar sulfonic acid group. The heteroatoms form covalent bonds with Fe 3d orbitals. But first, the negative charge on the polar head of SDS will facilitate columbic attraction to the anodic area. Therefore, there will be interference adsorption between SDS and aspartame on the anode. This may be the reason for the observed depreciating inhibition performance by the aspartame-SDS mixture relative to aspartame alone.

**Table 3:** Weight loss (WL), corrosion rate (*v*), surface coverage (θ), and inhibition efficiency (η_WL_) for T95 corrosion in 15 wt% HCl solution in the absence and presence of 6.80 mM aspartame in combination with 1 mM KI or 1 mM SDS or both at different temperatures from weight loss measurements.

Conc. (ppm)	WL ± S.D (g)	(*v* ± S.D) (mm/y)	θ	η_WL_%	WL ± S.D (g)	(*v* ± S.D) (mm/y)	θ	η_WL_%
	60 °C				70 °C			
Blank	0.15 ± 0.01	26.20 ± 0.17	–	–	0.35 ± 0.02	60.20 ± 3.47	–	–
ASP + KI	0.06 ± 0.01	10.41 ± 1.73	0.59	59	0.12 ± 0.02	20.82 ± 3.47	0.66	66
ASP + SDS	0.08 ± 0.01	13.88 ± 1.73	0.41	41	0.13 ± 0.01	22.55 ± 1.73	0.63	63
Formulation	0.05 ± 0.01	8.67 ± 1.73	0.70	70	0.07 ± 0.01	12.14 ± 1.73	0.79	79
	80 °C				90 °C			
Blank	0.61 ± 0.01	106.17 ± 1.73	–	–	0.86 ± 0.05	149.54 ± 8.67	–	–
ASP + KI	0.19 ± 0.01	32.96 ± 1.73	0.69	69	0.08 ± 0.01	13.88 ± 1.73	0.90	90
ASP + SDS	0.25 ± 0.02	43.37 ± 3.47	0.58	58	0.12 ± 0.01	20.82 ± 1.73	0.86	86
Formulation	0.06 ± 0.02	10.41 ± 3.47	0.87	87	0.08 ± 0.01	13.88 ± 5.20	0.91	91

ASP, aspartame; KI, potassium iodide; SDS, sodium dodecyl sulphate; Formulation = ASP + KI + SDS.

It is beneficial to rather mix aspartame, KI, and SDS. The iodide ions seem to have helped in stabilising both aspartame and SDS adsorptions. As could be seen in the table, the inhibition efficiency of the formulation is higher than that of the aspartame-KI mixture at all temperatures. The η_WL_ value recorded for the formulation at 60 °C, 70 °C, 80 °C, and 90 °C is 70%, 79%, 89%, and 91%, respectively. Besides the high η_WL_ value obtained for the formulation at the various temperature levels, it is worth drawing attention to the near constancy of the corrosion rate of the steel at all temperatures. The *v* is 8.67 ± 1.73 mm/y, 12.14 ± 1.73 mm/y, 10.41 ± 3.47 mm/y, and 13.88 ± 5.20 mm/y at 60 °C, 70 °C, 80 °C, and 90 °C despite reaching 149.54 ± 8.67 mm/y at 90 °C in the blank solution. This indicates that the formulation is effective and stable at high temperatures. Therefore, KI and SDS can be used to improve the adsorption stability of aspartame on steel at high temperatures.

##### EIS studies

**Figure 3:**
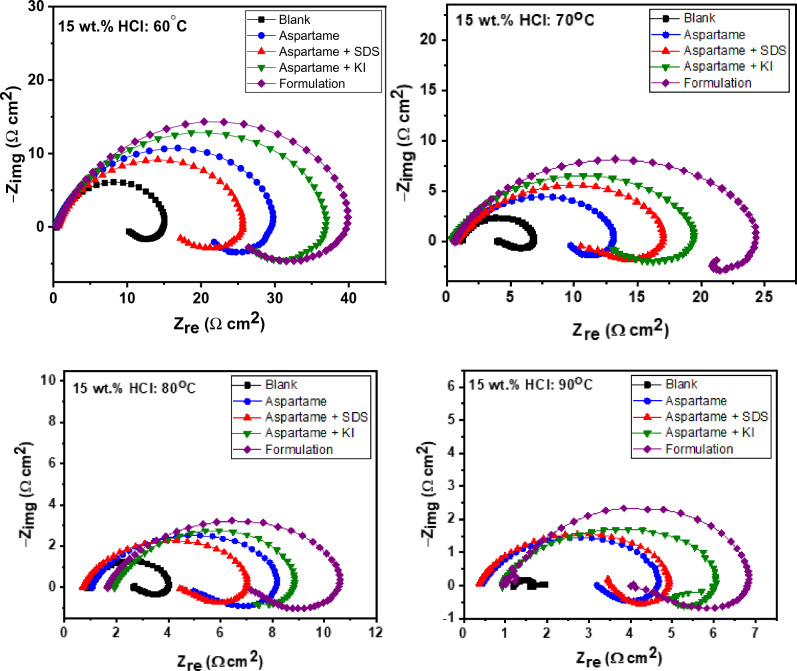
Nyquist electrochemical plots for T95 corrosion in 15 wt% HCl solution without and with 6.80 mM aspartame alone and in combination with 1 mM SDS or 1 mM KI or both (formulation) at different temperatures.

Figure 3 shows the Nyquist electrochemical diagrams for T95 corrosion in 15 wt% HCl solution without and with 6.80 mM aspartame alone and in combination with 1 mM KI or 1 mM SDS or both at 60–90 °C. The Nyquist diagrams drawn for the steel corrosion in the unprotected acid solution exhibit a capacitive loop at the high frequencies (HF) and an inductive loop at low frequencies (LF) at all temperatures except at 90 °C in which there is an additional poorly resolved capacitive loop at the middle frequencies (MF). A similar impedance characteristic was observed for pure iron^36,37^ in 1 M HCl solution and cold rolled steel in 0.5 M H_2_SO_4_ solution^38^. The HF semi-circular loop is often linked with the charge transfer of the dissolution process and double-layer behaviour^36–38^. The LF inductive loop is believed to result from the slow reaction during the adsorption of ${\mathrm{Cl}}_{\mathrm{ads}}^{-}$ and ${\mathrm{H}}_{\mathrm{ads}}^{+}$ on the working electrode surface^37^. It is also suggested that the re-dissolution of the passivated surface layer at LF could also give rise to an inductive loop^36–38^. The unresolved semi-circular loop at MF in the uninhibited Nyquist diagram at 90 °C is typical of Warburg impedance commonly associated with diffusion phenomenon^39^. It thus supports the assertion of aggravated corrosion of the steel at 90 °C earlier noted from the WL technique (Tables 1, 2). The corrosion products on the T95 steel surface, at 90 °C may have become too porous such that corrosive species were transported from the bulk solution to the steel/solution interface^39^. In the Nyquist diagrams drawn for the inhibited systems, the inductive loop is still seen but the MF capacitive loop at 90 °C is absent. This observation implies that the T95 steel electrode still corrodes by the direct charge-transfer process even in the presence of the additives but at 90 °C, the diffusion contribution to corrosion was abated. Overall, the inhibiting effect of the additives is demonstrated by the increase in the diameter of the capacitive loop relative to that of the blank. Similar to the observation made from the WL results (Table 2), the diameter of the HF capacitive loop increases in the order: formulation > aspartame + KI > aspartame > aspartame + SDS > blank at 60 °C and 80 °C. The diameter of the aspartame and aspartame + SDS graphs are almost the same at 90 °C. This indicates improved inhibition performance by the formulation followed by the aspartame-KI mixture and the antagonistic behaviour of SDS in the aspartame-SDS mixture. It should be mentioned that in all cases, the HF loops are not perfect semicircles. This phenomenon has always been attributed to the frequency dispersion resulting from the roughness and non-homogenous characteristics of the working electrode surface^38^.

**Figure 4:**
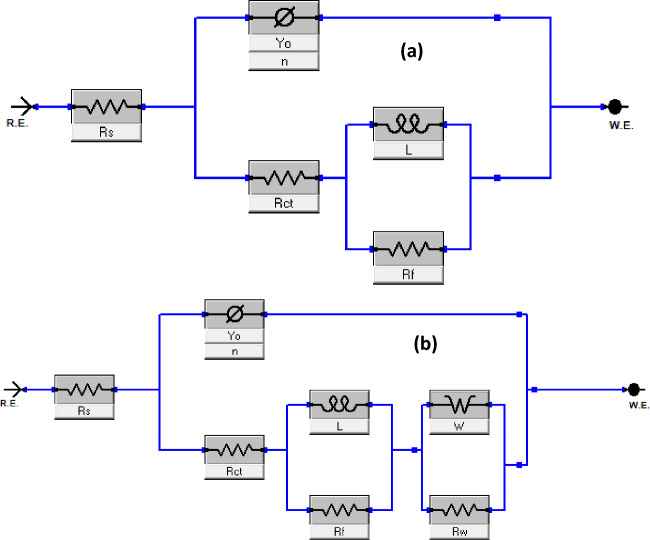
The equivalent circuit was used in the analysis of (**a**) all the electrochemical impedance data and (**b**) the data for blank at 90 °C.

The difference in the shape of the obtained impedance plot at 90 °C from others prompted the fitting of the impedance data with different equivalent circuits involving constant phase element (CPE), charge-transfer resistance (R_ct_), inductance (L), inductive resistance (R_f_), Warburg impedance (W), and Warburg resistance (R_W_). The equivalent circuits are shown in Fig. 4.

The double-layer capacitance (*C*_dl_) was calculated using the following equation^38^.

11 $${\mathrm{C}}_{dl}={Q}_{dl}\times {\left(2\pi {f}_{max}\right)}^{n-1}$$

where ${Q}_{dl}$ and *n* are the components of CPE and ${f}_{max}$ represents the frequency at which imaginary value reaches a maximum on the Nyquist plot^38^. The values for the fitted parameters are listed in Table 4. The inhibition efficiency (η_EIS_) was computed using Eq. (12)^24^.

12 $${\eta }_{\mathrm{EIS}}=\frac{{R}_{p(1)}-{R}_{p(0)}}{{R}_{p(1)}}\times 100$$

13 $${R}_{\mathrm{p}}={R}_{\mathrm{f}}+{R}_{\mathrm{w}}+{R}_{\mathrm{ct}}$$

where ${R}_{p(0)}$ and ${R}_{p(1)}$ are the polarization resistances in the absence and presence of additives, respectively^24^. As expected, the R_ct_, R_f_, and R_p_ values increase in the presence of additives while the C_dl_ value decreases indicating inhibitor adsorption on the steel surface and the mitigation of corrosion^40,41^. For all the studied inhibitors, η_EIS_ increases with increasing temperature due to the chemical adsorption of the inhibitor molecules^42^ and thus agrees with the WL results. The formulation produced the highest η_EIS_ of 92% at 90 °C. Contrary to the observation from WL studies (Table 3), the η_EIS_ for ASP + SDS marginally increase at 70 and 90 °C. Such a marginal increase is typical of competitive co-adsorption (antagonistic effect). As explained in Section “Studies on collective inhibition performance by additives with aspartame”, the polar head of SDS, chloride ions, and aspartame would compete for adsorption on the anodic region of the substrate. The competitive co-adsorption could at first result in a marginal increase in inhibition efficiency as suggested by the η_EIS_ at 70 and 90 °C. However, at prolonged immersion time due to the intensified molecules interactions, inhibition efficiency could slightly depreciate as noted in Table 3.

**Table 4:** EIS parameters were obtained for T95 corrosion in 15 wt% HCl at different temperatures without and with 6.80 mM aspartame alone and in combination with additives.

System	R_s_ ± Er (Ω cm^2^)	R_ct_ ± Er (Ω cm^2^)	R_f_ ± Er (Ω cm^2^)	L ± Er (Ω s cm^2^)	C_dl_ (µF cm^−2^)	*x*^2^ × 10^–3^	R_p_ ± Er (Ω cm^2^)	η_EIS_ %
	60 °C							
Blank	0.48 ± 0.01	9.98 ± 0.14	3.54 ± 0.15	10.50 ± 1.14	243	21.9	13.52 ± 0.29	-
ASP	0.42 ± 0.01	19.38 ± 0.50	5.93 ± 0.48	47.92 ± 9.11	131	53.5	25.31 ± 0.98	47
ASP + SDS	0.50 ± 0.01	17.54 ± 1.50	4.05 ± 0.50	22.58 ± 7.20	142	28.3	21.59 ± 2.00	37
ASP + KI	0.50 ± 0.01	21.73 ± 2.50	10.68 ± 2.71	35.81 ± 7.01	130	27.7	32.41 ± 5.21	58
Formulation	0.66 ± 0.01	24.54 ± 0.50	18.89 ± 0.50	52.63 ± 7.10	121	56.4	43.43 ± 1.00	69
	70 °C							
Blank	1.07 ± 0.01	3.38 ± 0.05	1.98 ± 0.06	2.68 ± 0.20	395	9.63	5.36 ± 0.11	-
ASP	0.77 ± 0.01	8.73 ± 0.14	2.14 ± 0.15	8.23 ± 1.42	238	34.0	10.87 ± 0.29	51
ASP + SDS	0.89 ± 0.01	10.25 ± 0.18	3.51 ± 0.18	14.83 ± 1.90	170	49.1	13.76 ± 0.36	61
ASP + KI	0.52 ± 0.01	12.30 ± 0.23	3.57 ± 0.23	11.36 ± 1.58	166	58.7	15.87 ± 0.46	66
Formulation	0.58 ± 0.01	19.26 ± 0.27	5.28 ± 0.30	11.36 ± 1.58	102	18.2	24.54 ± 0.57	78
	80 °C							
Blank	0.92 ± 0.01	1.89 ± 0.03	0.50 ± 0.03	0.85 ± 0.07	549	5.89	2.39 ± 0.06	-
ASP	1.14 ± 0.01	4.17 ± 0.07	2.13 ± 0.07	4.16 ± 0.36	253	15.1	6.30 ± 0.14	62
ASP + SDS	0.77 ± 0.01	3.96 ± 0.05	1.63 ± 0.06	2.67 ± 0.26	236	17.7	5.59 ± 0.11	57
ASP + KI	2.01 ± 0.01	5.57 ± 0.28	1.82 ± 0.26	1.98 ± 0.64	225	15.9	7.39 ± 0.54	68
Formulation	1.80 ± 0.00	8.71 ± 0.09	4.24 ± 0.10	3.92 ± 0.44	251	11.8	12.95 ± 0.19	82
	90 °C							
Blank	1.20 ± 0.01	0.44 ± 0.02	0.20 ± 0.10 0.05 ± 0.01^a^	0.80 ± 0.05	439	0.92	0.69 ± 0.19	-
ASP	0.68 ± 0.01	3.92 ± 0.06	0.61 ± 0.06	0.96 ± 0.24	266	16.4	4.53 ± 0.12	85
ASP + SDS	0.78 ± 0.00	4.22 ± 0.05	1.21 ± 0.10	1.12 ± 0.12	276	18.4	5.43 ± 0.15	87
ASP + KI	0.63 ± 0.01	3.38 ± 0.05	2.98 ± 0.04	1.08 ± 0.20	246	22.7	6.36 ± 0.09	89
Formulation	0.83 ± 0.01	5.04 ± 0.43	3.72 ± 0.04	11.20 ± 0.90	204	12.80	8.76 ± 0.47	92

^a^*R*_*w*_ value; ASP, Aspartame; KI, potassium iodide; SDS, sodium dodecyl sulphate; Formulation = ASP + KI + SDS.

##### PDP studies

**Figure 5:**
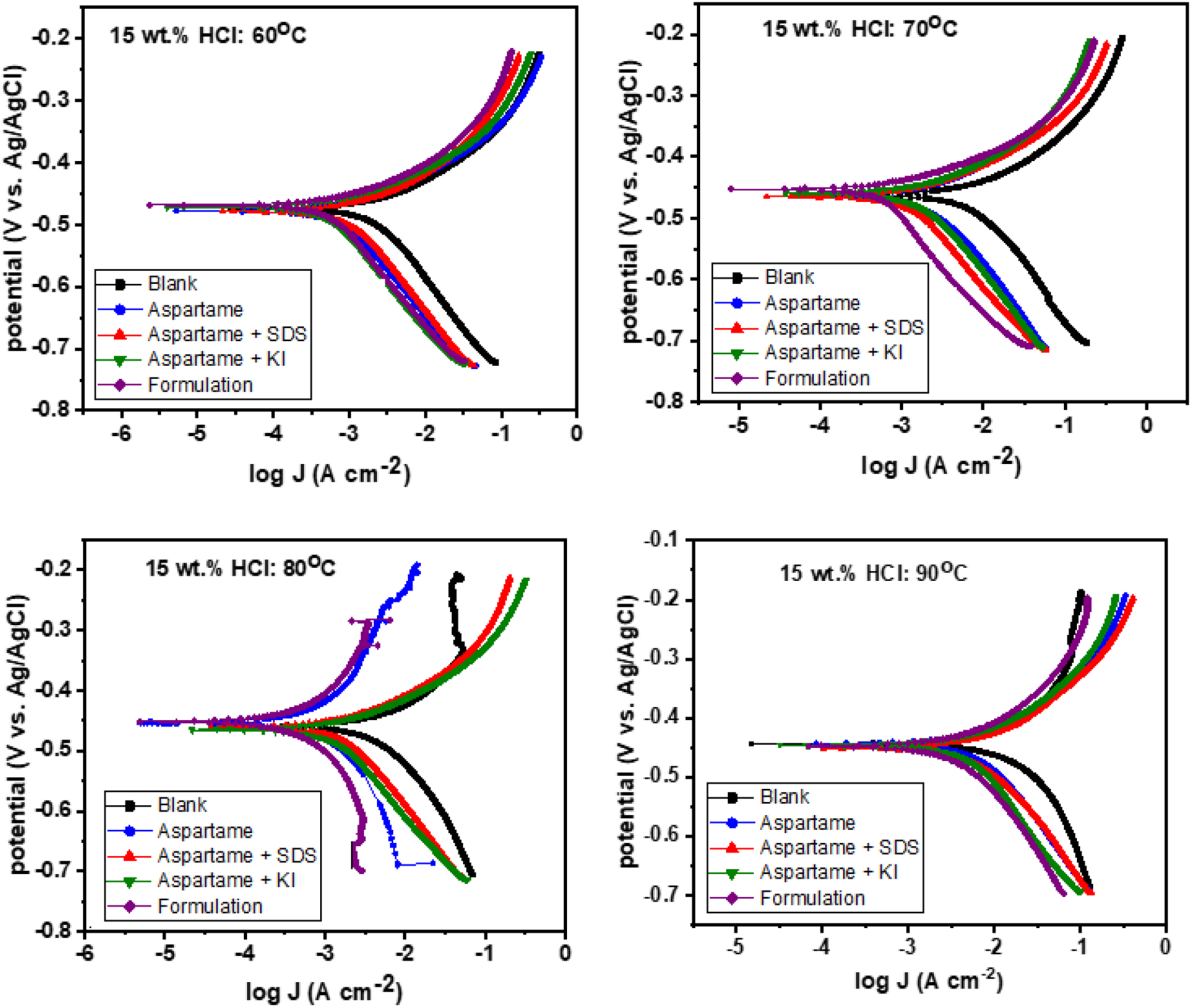
Potentiodynamic polarization plots for T95 corrosion in 15 wt% HCl solution without and with 6.80 mM aspartame alone and in combination with 1 mM SDS or 1 mM KI or both (formulation) at different temperatures.

To further evaluate the influence of the addition of SDS or KI or both on the inhibition performance of aspartame, polarization measurements were performed. Figure 5 displays the PDP plots for T95 corrosion in 15 wt% HCl solution without and with 6.80 mM aspartame alone and in combination with 1 mM SDS or 1 mM KI or both (formulation) at 60 °C, 70 °C, 80 °C, and 90 °C. The values of the polarization parameters including the corrosion potential (*E*_corr_), *i*_corr_, and anodic and cathodic slopes (*β*_a_ and* β*_c_) are given in Table 5. The corrosion rate ($v$) and η_PDP_ were computed using Eqs. (14) and (15)^43^, respectively.

14 $$v=\frac{K\times {i}_{\mathrm{corr}}\times EW}{\rho }$$

15 $${\eta }_{\mathrm{PDP}}=\frac{{i}_{corr}^{0}-{i}_{corr}^{inh}}{{i}_{corr}^{0}}\times 100$$

where $K$ is the conversion constant (3.27 × 10^−3^ mm g/μA cm y when $v$ is in mm/y), $\rho$ is density in g/cm^3^, $EW$ is the equivalent weight, ${i}_{corr}^{0}$ and ${i}_{corr}^{inh}$ are the corrosion current densities in the absence and presence of inhibitor. The presence of the additives is seen to cause both the anodic and cathodic curves to shift in the direction of low current density at all temperatures. The trend is as noted in other techniques, that is, the formulation produced the most effect. The results in Table 5 reveal that the ${i}_{corr}^{0}$ increases with the rise in temperature reaching a value of 16,100 μA cm^−2^ at 90 °C. Consequently, a startling corrosion rate of 186.37 mm/y is obtained at 90 °C confirming the aggravated corrosion at 90 °C that the results from other techniques (Tables 1, 2, Fig. 3) suggested. The remarkable reduction of *v* from 186.37 mm/y at 90 °C to 14.35 mm/y corresponding to an inhibition efficiency of 92% by the formulation is worth noting. One of the key requirements for an acidizing oil well inhibitor is thermal stability and effectiveness retention at high temperatures. The results imply that the formulation could satisfactorily restrain the corrosion of oil well tubing during acidizing operation. It is obvious from the polarization curves that the aspartame mixture exhibits a mixed-type inhibiting behaviour. The insignificant displacement of the *E*_corr_ in the presence of the additives and the simultaneous reduction of the anodic and cathodic current densities attest to the mixed-type behaviour^44^. However, it could be seen that there is suppression dominance of the cathodic half-reduction reaction especially at 60 °C and 70 °C (Fig. 3). In Table 5, the *β*_c_ values are seen to be much bigger than the *β*_a_ values. This is reasonable since the protonated form of aspartame is expected to adsorb at the cathodic region to suppress the adsorption of ${\mathrm{H}}_{\mathrm{ads}}^{+}$ and the subsequent evolution of hydrogen gas. The inhibition of the anodic oxidation reaction will very much depend on the recharging power of the additives. The PDP and the EIS results are in good agreement.

**Table 5:** Potentiodynamic polarization parameters derived for T95 corrosion in 15 wt% HCl at different temperatures without and with 6.80 mM aspartame alone and in combination with 1 mM KI or 1 mM SDS or both.

System	$-$ *E*_corr_ (mV vs. Ag/AgCl)	*i*_corr_ (μA cm^−2^)	β_a_ (V dec^−1^)	$-$ β_c_ (V dec^−1^)	*v* (mm/y)	η_PDP_ %
	60 °C					
Blank	474.0	2240	62.30	173.40	25.93	-
ASP	478.0	1160	49.90	151.90	13.43	48
ASP + SDS	478.0	1060	57.20	164.80	12.27	52
ASP + KI	472.0	890	45.30	161.80	10.30	60
Formulation	469.0	590	49.10	165.30	6.83	74
	70 °C					
Blank	477.0	5270	135.90	531.20	61.00	-
ASP	376.0	2050	81.80	160.80	23.73	61
ASP + SDS	465.0	1760	55.40	174.50	20.37	67
ASP + KI	460.0	1720	66.90	179.20	19.91	67
Formulation	453.0	1000	41.20	178.30	11.58	81
	80 °C					
Blank	460.0	9500	22.90	133.70	109.97	-
ASP	459.0	3380	90.00	200.00	39.13	64
ASP + SDS	460.0	4340	67.20	178.90	50.24	54
ASP + KI	465.0	3290	56.90	176.60	38.08	65
Formulation	451.0	1850	49.60	164.00	21.41	83
	90 °C					
Blank	443.0	16,100	154.00	209.00	186.37	-
ASP	448.0	2160	100.00	210.00	25.00	87
ASP + SDS	451.0	2010	88.90	190.90	23.27	88
ASP + KI	443.0	1500	93.20	218.90	17.36	91
Formulation	446.0	1240	91.70	251.80	14.35	92

ASP, aspartame; KI, potassium iodide; SDS, sodium dodecyl sulphate; Formulation = ASP + KI + SDS.

### Synergism studies and explanation of inhibition mechanism

Synergism and antagonism are the two terms often used to describe the nature of adsorption in a binary or multiple-component system. By definition, synergistic effect implies the cooperative co-adsorption of the participating species on a substrate surface while antagonistic effect means the separate co-adsorption of the species^45^. The synergism parameter (*S*) since used by Aramaki and Hackerman^46^ has been overwhelmingly applied in classifying co-adsorption as synergistic or antagonistic^34,47,48^. By the $S$ classification, a $S>1$ indicates a synergistic effect and $S<1$ means an antagonistic effect^45^. Most recently, Kokalj^49^ proposed the computation of $S$ from the corrosion activity ($\alpha$) and the threshold corrosion activity (${\alpha }^{\mathrm{threshold}}$) of an inhibitor following Eq. (16). The $\alpha$ can be obtained using Eq. (17). Equation (18) is used for the calculation of ${\alpha }^{\mathrm{threshold}}$ for a binary system or ternary system^49^.

16 $$S=\frac{{\alpha }^{\mathrm{threshold}}}{{\alpha }_{1+2}} \mathrm{or}\frac{{\alpha }^{\mathrm{threshold}}}{{\alpha }_{1+2+3}}$$

17 $$\alpha =1-\uptheta$$

18 $${\alpha }^{\mathrm{threshold}}=\frac{{\alpha }_{1}{\alpha }_{2}}{{\alpha }_{1}+{\alpha }_{2}} \mathrm{or}\frac{{\alpha }_{1}{\alpha }_{2}{\alpha }_{3}}{{\alpha }_{1}{\alpha }_{2}+{\alpha }_{1}{\alpha }_{3}+{\alpha }_{2}{\alpha }_{3}}$$

**Figure 6:**
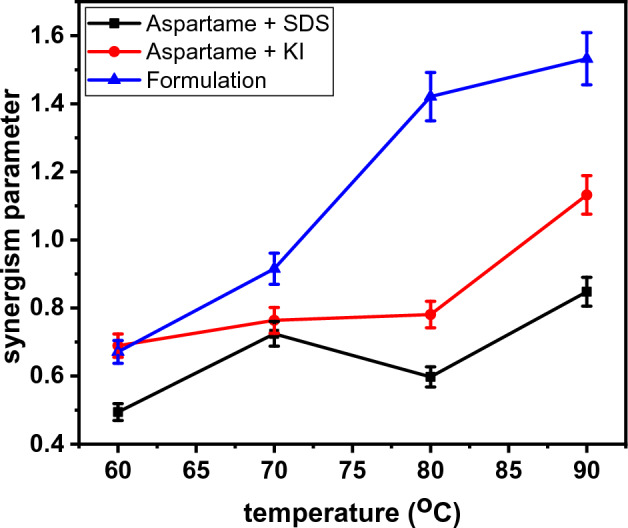
Variation of synergism parameter with temperature.

In this work, $\uptheta$ is the surface coverage obtained using the inhibition efficiency value from the EIS, *i.e*., $\uptheta =\frac{\mathrm{\eta EIS}}{100}$. ${\alpha }_{1},{\alpha }_{2},$ and ${\alpha }_{3}$ are the corrosion activity of aspartame, KI, and SDS, respectively. The plot of $S$ as a function of temperature is shown in Fig. 6.

For the aspartame-SDS system, $S$ is less than one at all temperatures implying an antagonistic effect between aspartame and SDS. This result is reasonable, agrees with the results from the PDP studies (Fig. 5), and can be explained thus.

1. In the blank acid solution (15 wt% HCl), Fe oxidation takes place at the anode ($\mathrm{Fe }\to {\mathrm{Fe}}^{2+}+{2e}^{-}$) and hydrogen ions reduction occurs at the cathode (2H^+^ + 2*e*^−^ → H_2_)^50^. Chloride ions (${\mathrm{Cl}}^{-}$) would be attracted to the positive anodic area and would be adsorbed on the metal surface making the anodic area partially negative.
2. In the acid solution containing aspartame, the amino group in aspartame is expected to gain a proton which would make aspartame exist in cationic form and adsorbable at the cathode. Such adsorption would lead to the suppression of the cathodic hydrogen reduction reaction and the PDP results (Fig. 5) attest to this. Some of the protonated aspartame would be attracted to the partially recharged anodic area through coulombic attraction. On the anode, by donation of electron pair on the unprotonated heteroatoms to the d-orbital of Fe, chemical bonding takes place. The variation of the inhibition efficiency with temperature alludes to the existence of chemisorption.
3. In the acid solution containing SDS, because of the negative sulphate head, the surfactant would be attracted to the anode and would be in contention of adsorption with chloride ions. As earlier mentioned, the electron pair on the heteroatoms would be donated to the d-orbital of Fe to ensure a covalent type of bonding. Therefore, when aspartame and SDS are mixed, both would compete for adsorption at the anodic area making the anodic inhibition diminish and the aspartame molecules adsorbed on the cathodic area to be the main contributor to the inhibition process. This can be seen in Fig. 5. It also explains the reason behind the lower inhibition efficiency value of the aspartame-SDS mixture relative to aspartame alone (Table 4).

Figure 6 reveals an antagonistic behaviour between KI and aspartame at 60 °C, 70 °C, and 80 °C but a synergistic effect at 90 °C. Unlike the aspartame-SDS system, the *S* value for the aspartame-KI mixture is very close to unity meaning better synergy between aspartame and KI than SDS. Aramaki and Hackerman^46^ reported a similar antagonistic effect between iodide ions and cyclic imine and attributed it to interference in the adsorption process at the anode. Iodide ions, as explained in section “Studies on individual inhibition performance by additives” are preferentially adsorbed on the anode at the expense of chloride ions^51^. The higher recharging power of iodide ions facilitates the attraction of more aspartame cations onto the anodic region. Aramaki and Hackerman^46^ explained that a high concentration of I^−^ ions (in other words, a thick adsorbed iodide ion layer) could interfere with the electron pair donation and acceptance process on the anode. This may have been the case at 60–80 °C. At 90 °C, because a significant amount of I^−^ ions have been oxidized to ${\mathrm{I}}_{3}^{-}$ ions, interference of the adsorbed I^−^ ions with the donation and acceptance process between aspartame and Fe on the anode may have been reduced while the stabilization by diminishing the coulombic repulsion between the cationic aspartame and the charged steel surface enhances their synergism.

In the formulation system, a strong synergistic effect is observed at 80 °C and 90 °C. Individual dominance may have been minimal in the ternary system which ensured their cooperative co-adsorption resulting in the observed high inhibition performance.

### Surface observation

#### SEM and EDX studies

**Figure 7:**
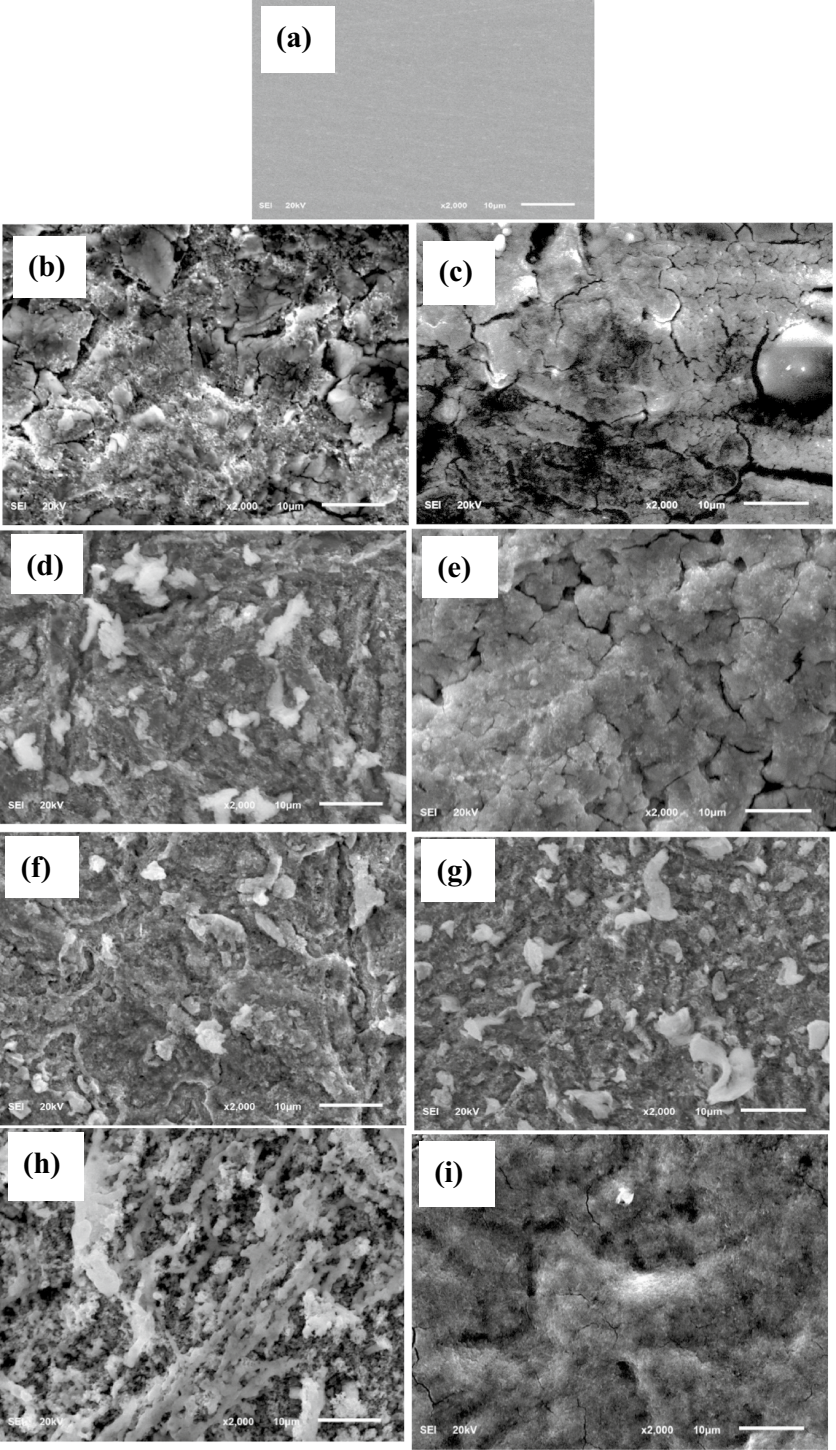
SEM micrographs of abraded of T95 (**a**) before and after immersion in 15% HCl solution (**b**, **c**) without inhibitor, (**d**, **e**) containing 6.80 mM aspartame, (**f**, **g**) containing 6.80 mM aspartame + 1 mM KI, (**h**, **i**) containing 6.80 mM aspartame ASP + 1 mM KI + 1 mM SDS (formulation) at 60 °C (**b**, **d**, **f**, **h**) and 90 °C (**c**, **e**, **g**, **i**) for 4 h.

**Figure 8:**
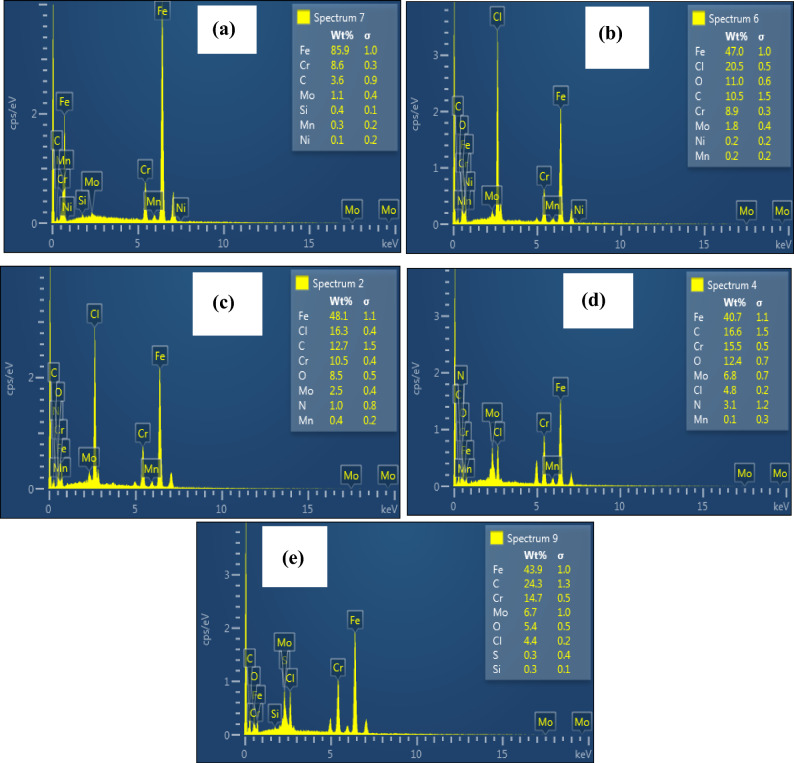
EDX spectra of abraded T95 (**a**) before and after immersion in 15 wt% HCl solution (**b**) without inhibitor, (**c**) containing 6.80 mM aspartame, (**d**) containing 6.80 mM aspartame + 1 mM KI, and (**e**) containing 6.80 mM aspartame + 1 mM KI + 1 mM SDS (formulation) at 90 °C for 4 h.

The surface of the T95 steel specimen before and after immersion in 15 wt% HCl solution without and with 6.80 mM aspartame alone and in combination with 1 mM KI or 1 mM KI + 1 mM SDS at 60 °C and 90 °C for 4 h was analyzed with SEM and EDX. The SEM micrographs of the T95 sample before and after corrosion at 60 °C and 90 °C are shown in Fig. 7. The composition of the steel surface products at 90 °C from EDX analysis is displayed in Fig. 8. Figure 7a exemplifies the smooth surface morphology of the T95 steel sample after mechanical abrasion which based on Fig. 8a has 85.9 wt% Fe, 8.6 wt% Cr, 3.6 wt% C, 1.1 wt% Mo, 0.4 wt% Si, 0.3 wt% Mn, and 0.1 wt% Ni in conformity with the chemical composition of T95-2 steel grade^12^. The steel underwent severe corrosion in the acid solution that resulted in surface deformation (Fig. 7b,c). Heaps of porous and cracked corrosion product is seen on the surfaces shown in Fig. 7b,c. Relative to the EDX spectrum in Fig. 8a,b, reveals the presence of a significant amount of Cl (20.5 wt%), an increase in the wt% of O and C to 11.0 and 10.5, respectively and a decrease in the wt% of Fe to 47.0. This observation implies that the corrosion product is a mixture of chlorides, oxides, hydroxides, and carbonates (Fig. 8b) which aligns with previous reports^45^. The presence of N and the increase in the wt% of C (12.7) in Fig. 8c compare to Fig. 8b provide experimental evidence that the product in Fig. 7d,c is adsorbed aspartame molecules. The significant decrease in the wt% of Cl from 20.5 (Fig. 8b) to 16.3 (Fig. 8c) indicates corrosion inhibition by the adsorbed aspartame molecules. However, an inspection of Fig. 7d, c reveals that aspartame alone could not satisfactorily protect the steel surface, especially at 90 °C. A worn-out and cracking surface morphology is seen in Fig. 7c. In agreement with the other experimental results (Tables 3, 4, 5), the addition of KI enhanced the inhibiting ability of aspartame but adding KI and SDS to aspartame benefitted the inhibiting property of aspartame the most, especially at 90 °C. As could be seen in Fig. 7f–i, the adsorbed surface product is compact and more evenly distributed at 90 °C. Figure 8d,e reveal a substantial decline in the Cl concentration on the aspartame-KI (decreased to 4.8 wt%) and the formulation (decreased to 4.4 wt%) protected surfaces which again confirm the effectiveness of the formulation as an acidizing corrosion inhibitor.

#### OP studies

**Figure 9:**
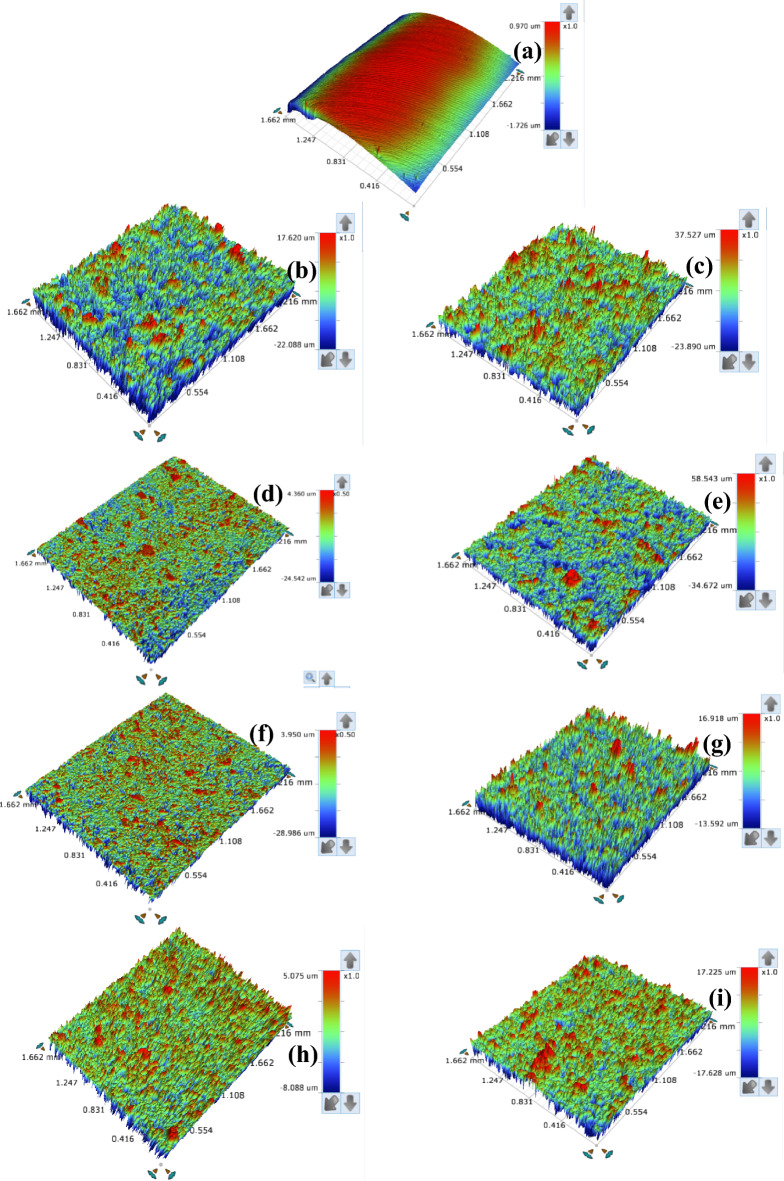
Surface profilometer images of abraded T95 steel (**a**) before and after immersion in 15 wt% HCl solution (**b**, **c**) without inhibitor, (**d**, **e**) containing 6.80 mM aspartame, (**f**, **g**) containing 6.80 mM aspartame + 1 mM KI, (**h**, **i**) containing 6.80 mM aspartame + 1 mM KI + 1 mM SDS (formulation) at 60 °C (**b**, **d**, **f**, **h**) and 90 °C (**c**, **e**, **g**, **i**) for 4 h.

Figure 9 shows the OP images of abraded T95 steel (a) before and after immersion in 15 wt% HCl solution (b,c) without inhibitor, (d,e) containing 6.80 mM aspartame, (f,g) containing 6.80 mM aspartame + 1 mM KI, (h,i) containing 6.80 mM aspartame + 1 mM KI + 1 mM SDS (formulation) at 60 °C (b,d,f,h) and 90 °C (c,e,g,i) for 4 h. Generally, the surface property can be quantified using roughness parameters. Commonly used roughness parameters are the average roughness (R_a_), root mean square roughness (R_q_), and the total height of profile (R_t_) and give information on the irregularity of deviation from an ideal smooth surface^52^. The value of R_a_, R_q_, and R_t_ for the studied steel surface is given in Table 6.

**Table 6:** Surface parameters derived from profilometer surface analysis of the corroded T95 immersed in 15 wt% HCl without and with additives at 60 °C and 90 °C after 4 h of immersion.

Systems/concentration	Surface roughness					
	60 °C			90 °C		
	R_a_ (µm)	R_q_ (µm)	R_t_ (µm)	R_a_ (µm)	R_t_ (µm)	R_t_ (µm)
Abraded T95	0.154	0.186	2.119	0.154	0.186	2.119
Blank	2.269	2.933	39.708	4.350	5.679	61.417
ASP	0.925	1.170	28.902	3.736	5.029	93.215
ASP + KI	0.923	1.155	32.936	2.279	2.992	30.510
Formulation	0.435	0.155	4.204	0.550	1.599	14.853

ASP, aspartame; KI, potassium iodide; Formulation = ASP + KI + SDS.

R_a_, R_q_, and R_t_ value of 0.154 µm, 0.186 µm, and 2.119 µm, respectively was recorded for the smooth abraded surface in Fig. 9a. The rougher surface after corrosion in the blank acid solution can be picturized in Fig. 9b,c. The R_a_, R_q_, and R_t_ values increase by 93% and 96% at 60 °C and 90 °C, respectively. For instance, the R_a_ value increases from 0.154 to 2.269 and 4.350 µm at 60 °C and 90 °C, respectively. The surfaces in Fig. 9f–i are smoother compared to the surfaces in Fig. 9b,c due to the adsorption of the inhibitors and the protection of the surface against corrosion. It is again worth emphasizing the outstanding inhibitive performance of the formulation at 90 °C. As could be seen in Fig. 9i, the surface is very smooth when compared to the aspartame-KI surface in Fig. 9g. In Table 6, the R_a_ value is seen to decrease from 4.350 µm at 90 °C to 0.550 µm for the formulation-inhibited surface corresponding to a 79% reduction. It is therefore concluded that the formulation is highly effective against the corrosion of T95 steel in 15 wt% HCl solution at high temperatures.

## Summary and conclusions

The inhibiting ability of aspartame, a natural sweetener in combination with potassium iodide and sodium dodecyl sulphate was explored against the corrosion of a typical oil well tubing material (T95-2 grade) in 15 wt% HCl solution at 60–90 °C. The weight loss, electrochemical (EFM, EIS, PDP) and surface analysis (SEM, EDX, OP) techniques were used in the investigation. The findings from the investigation are summarized as follows.

1. T95 steel undergoes serious corrosion in 15 wt% HCl solution and the corrosion rate can reach 186.37 mm/y at 90 °C.
2. Aspartame exhibits a corrosion inhibiting effect against T95 steel corrosion in 15 wt% HCl solution and its effect increases with an increase in temperature. A concentration of 6.80 mM can afford an inhibition efficiency of 86% at 90 °C.
3. A 1 mM concentration of potassium iodide and sodium dodecyl sulphate exhibits better inhibiting ability than 3- and 5-mM concentrations.
4. Addition of 1 mM concentration of sodium dodecyl sulphate to 6.80 mM aspartame produce an antagonistic effect. KI-aspartame mixture exhibits an antagonistic effect at 60 °C and 70 °C but a synergistic effect at 80 °C and 90 °C.
5. It is beneficial to mix sodium dodecyl sulphate (1 mM), potassium iodide (1 mM), and aspartame (6.80 mM). The mixture is capable of reducing the corrosion rate of T95 steel in 15 wt% HCl solution from 186.37 mm/y to 14.35 mm/y and protecting the surface by 92% at 90 °C.
6. The mechanism of inhibition by aspartame alone and in combination with KI and sodium dodecyl sulphate is adsorptive. It inhibits both anodic and cathodic corrosion reactions.

## Data Availability

The datasets used and/or analyzed during the current study available from the corresponding author on reasonable request.
